# Hospital Festivals, Meetings, &c.

**Published:** 1894-04-14

**Authors:** 


### HOSPITAL FESTIVALS, MEETINGS, ETC.

### HOSPITAL FOR DISEASES OF THE THROAT, GOLDEN SQUARE.

The annual meeting of governors and subscribers was held on April 5th at 20, Hanover Square. The annual report gave the number of in-patients for 1893 as 526, and of out-patients as 7,907, the numbers being practically identical with those of the previous year, in spite of the fact that the building was closed for several weeks last summer. The adoption of the report was moved by the chairman, the Duke of Sutherland. He was followed by Mr. Courtenay Welch, who spoke of the loss the institution had sustained in the death of Lord Calthorpe, whose place as president the Duke of Sutherland had consented to take. Mr. Welch also spoke of the urgent need for an extension of the hospital, the women's wards especially being always full; but said that only about £3,000 of the necessary £5,000 was at present available for the purpose. Mr. Mark Hovell, in acknowledging the vote of thanks to the medical staff, said that it was a source of gratification to them to know that the hospital was visited, for the purposes of study, by men from all over the world He also bore witness to the cordial relations subsisting between the medical staff and the committee of management. Among other speakers were Mr. Alfred Mackenzie, Dr. Greville Macdonald, and Mr. Haslehurst.

### CLERGY ORPHAN SCHOOLS.

If the claims of an institution on the benevolent are measured by its usefulness and the need for its existence, there are few whose appeal for support is so well founded as that of the Clergy Orphan Schools. No profession is so ill-paid as the parson's, and of none is so much expected. The stipend of five out of six of the clergy is not more than from £150 to £200 a year. On this pittance they are supposed to keep up the appearance of gentlemen, and have constantly to put their hands in their pockets to relieve distress. The general impressionis, it is true, that they obtain funds for their charitable work by putting their hands into other people's pockets, but certainly they do not, as a rule, spare their own. Imprudent as it may be, they naturally fall in love and marry, as other folks do, and a not unusual consequence is that "olive branches" gather round their table. Then begins a hand to mouth struggle which lasts as long as life lasts. Hard as it is to bring up children on such an income, if the husband dies the income ceases, and wife and children often stand face to face with utter destitution. In this emergency the help held out by the Clergy Orphan Schools is a God-send gift to many a poor widow's aching heart. Boys and girls are received into the schools, fed, clothed, educated thoroughly, and started afterwards in life, at no cost to the widow. The education given is excellent, and such as it is fitting the children of ladies and gentlemen should receive. The income of the corporation is £15,000 a year, of which £6,250 is derived from subscriptions and the remainder from invested funds. With this sum about three hundred boys and girls are provided for in the fine schools at Canterbury and St. John's Wood respectively. Considering what is done for the boys and girls and the first-rate education that is given, £50 a head yearly is a very moderate expenditure, and evidently the funds are prudently and economically administered. The working expenses amount to 9 per cent., or one pound only out of eleven of the money spent. This compares favourably with the expenditure of institutions of a similar character, and it is most satisfactory to see so excellent a charity managed in every way so well as the Clergy Orphan Schools are. The pity is that funds do not allow of their admitting the hundreds of cases which have to appeal to them in vain.

### LONDON HOMŒOPATHIC HOSPITAL.

The report presented to the Governors of this hospital at the annual general meeting on April 10th was confined to the nine months ending December 31st, 1893, in accordance with the determination arrived at by the Board to close their financial year in future in December instead of March. The number of in-patients during this period was 445, as against 557 in the nine months ending December 31st, 1892, the decrease in numbers being due to the limited accommodation — forty beds only — in the temporary hospital, in which the work of the institution is carried on until the completion of the new buildings. These are expected to be completed in May, 1895; and, in order to open them free of debt, the sum of £12,000 is still required. The chairman, Mr. T. Pakenham Stilwell, in moving the adoption of the report, referred to the death of Lord Ebury, the president of the hospital; and it was afterwards proposed and carried that the Earl of Wemyss and March should be asked to accept the office. Among the other speakers were Dr. Clifton, of Northampton, who urged that the practice of visiting patients at their own homes should be still further extended, in view of the beneficial results which such visits had produced in Liverpool and other places; the Rev. Dacre Craven, chaplain to the hospital, Dr. Dudgeon, Dr. Dyce Brown, and Mr. Knox Shaw. At the conclusion of the general meeting, a special general meeting was held for the purpose of requesting the governors and subscribers to authorize the trustees to appropriate £2,000 of the Reserve Fund towards defraying the necessary expenses incurred in connexion with the temporary hospital. The motion having been carried unanimously, the chairman expressed a hope that sufficient funds would before long be raised to enable them to pay back what they should continue to regard as a debt to the Reserve Fund.

